# Arthroscopic Anterior and Posterior Glenohumeral Capsular Augmentation With Gracilis Allograft

**DOI:** 10.1002/atn2.70017

**Published:** 2026-04-28

**Authors:** Michael D. Maloney, Omkar N. Prabhavalkar, Samantha Levin, Richard Lander, Brett P. Salazar, Sandeep Mannava

**Affiliations:** ^1^ Department of Orthopaedics & Physical Performance Divisions of Sports Medicine, Shoulder and Elbow Surgery University of Rochester Medical Center Rochester New York U.S.A.

## Abstract

Multidirectional instability in patients with connective tissue disorders such as Ehlers‐Danlos syndrome (EDS) is difficult to treat due to poor tissue quality and frequent failure of standard repairs. We describe an arthroscopic technique using gracilis tendon allografts to perform an anterior and posterior capsular augmentation in the revision setting with subscapularis deficiency. Allografts are anchored to the glenoid and humeral head in a sling configuration, restoring capsular restraint and stability. This method builds upon prior posterior‐only augmentation by providing circumferential support and utilizing allograft to overcome compromised native tissue.

## Graphical abstract

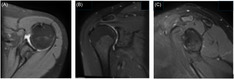

**VIDEO 1:** Anterior and posterior capsular augmentation technique performed in the left shoulder. With the patient in the lateral decubitus position, and through the use of both anterior and posterior viewing portals, two allografts, 65 mm and 55 mm, are anchored posteriorly and anteriorly, respectively, each using two 2.9‐mm PushLock suture anchors to the glenoid surface and one 4.75 mm SwiveLock anchor to the humeral head. Video content can be viewed at https://doi.org/10.1002/atn2.70017.

Shoulder instability may result from traumatic injuries, repetitive motion, or connective‐tissue disorders such as Ehlers‐Danlos syndrome (EDS). In cases involving connective‐tissue disorders such as EDS, surgical management is challenging, especially when compounded by previous failed surgeries. Surgical options for patients with EDS with multidirectional instability include arthroscopic versus open capsulorrhaphy with pancapsular shift.^1^, ^2^, ^3^, ^4^, ^5^ Thermal capsulorrhaphy is a historical option that has been largely abandoned due to complications including chondrolysis and high rates of failure.^6^, ^7^, ^8^ The open capsular shift is regarded as one of the gold‐standard procedures for treatment of multidirectional instability.^9^ Further, open graft augmentation of the joint capsule and rotator cuff are other surgical options available.^10^, ^11^ Latarjet and other bone block procedures can be useful in aiding shoulder stability through bony augmentation with promising outcomes.^12^, ^13^ Latarjet and distal tibial allograft are typically reserved for patients with glenoid bone loss.^14^, ^15^, ^16^

In patients with underlying soft‐tissue disorders such as EDS, the disordered collagen leads to increased laxity and high risk of instability. These patients are also at high risk for recurrent instability following surgical intervention due to their underlying condition. Due to the compromised soft tissue in these individuals, use of allografts may provide a longer‐lasting solution than working with the patient's native tissue. One possible surgical intervention is a posterior capsular augmentation with gracilis allograft to address recurrent instability and recreate a capsular “sling” to improve shoulder stability for the patient.^17^

In patients with EDS who have had multiple previous procedures or chronic subscapularis deficiency, an anterior capsular augmentation combined with a posterior capsular augmentation may also be beneficial. The technique described here uses 2 portions of a gracilis allograft to perform an anterior and posterior capsular augmentation for multidirectional stability in the revision shoulder surgery setting for a patient with EDS with subscapularis deficiency.

## SURGICAL TECHNIQUE

### Indications

This technique is indicated for patients with multidirectional shoulder instability in the setting of connective tissue disorders such as EDS, particularly those with poor capsular tissue quality, chronic subscapularis insufficiency, or a history of failed prior stabilization procedures. These patients often present with instability and inadequate soft‐tissue integrity, which limit the effectiveness of other approaches such as open subscapularis repair, especially due to scarring from prior surgeries. In such cases, an anterior and posterior capsular augmentation through a sling configuration using gracilis tendon allograft may provide improved joint stability.

**FIGURE 1:**
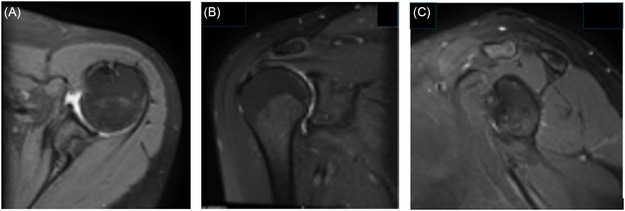
Preoperative magnetic resonance images (A) Left shoulder, axial magnetic resonance image with posteriorly subluxated humeral head and subscapularis deficiency. (B) Left shoulder, coronal magnetic resonance image with intact supraspinatus tendon. (C) Left shoulder, sagittal magnetic resonance image showing anchors from prior open Bankart repair and capsulorrhaphy.

A demonstration of the surgical technique in a left shoulder is provided in Video 1. Preoperative magnetic resonance imaging of the left shoulder shows intact supraspinatus tendon and subscapularis deficiency (Figure 1A‐C).

#### Patient Positioning and Preparation

Under general anesthesia, the patient is placed lateral decubitus with the arm abducted 50°, flexed 20°, and in balanced suspension.

#### Diagnostic Arthroscopy

Standard posterior and anterior, mid‐glenoid arthroscopic portal are established, 2 cm distal and medial to the posterolateral corner of the acromion and just lateral to the coracoid, respectively. A diagnostic arthroscopy is conducted from the posterior portal, examining the soft‐tissue structures and bony anatomy of the glenohumeral joint. Any indicated labrum repairs would be undertaken before proceeding with the remainder of the procedure.

#### Graft Preparation

**FIGURE 2:**
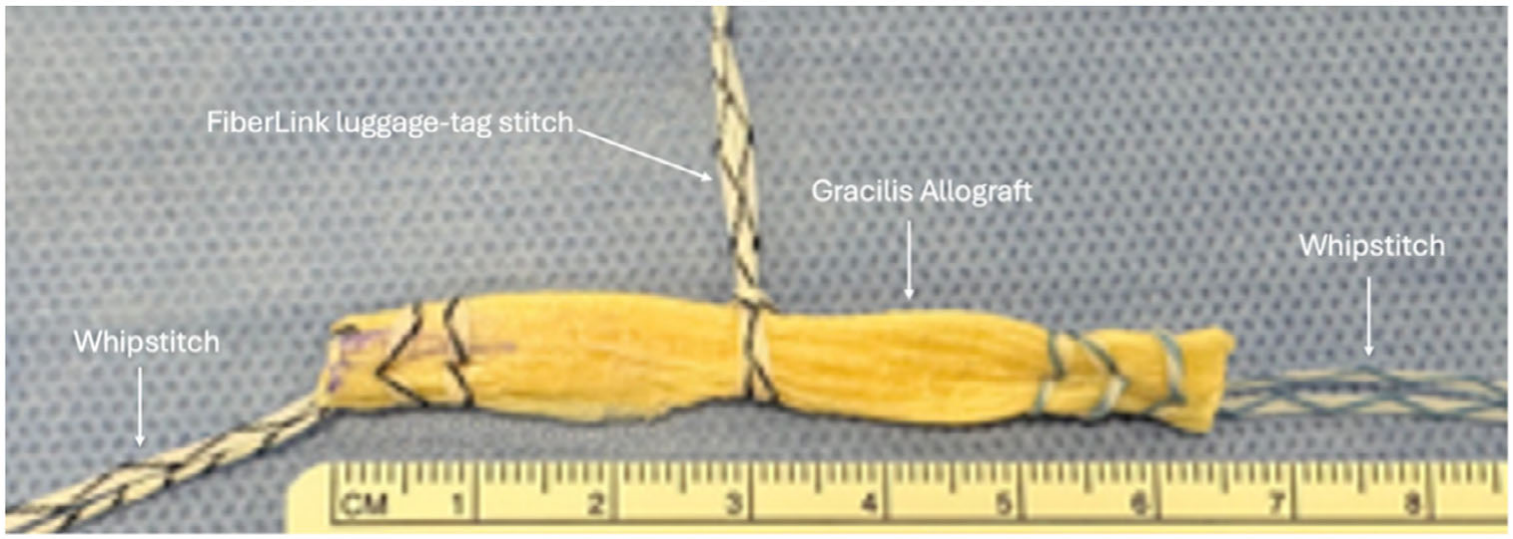
Left shoulder with the patient in the lateral decubitus position. The gracilis tendon allograft, trimmed to 6.5 cm in length, with a single suture (FiberLink; Arthrex) in luggage‐tag configuration at the midpoint of the graft, and whipstitches (FiberLoop; Arthrex) at the 2 ends of the graft in different colors.

Preparation of the graft for posterior capsular augmentation begins by selecting a gracilis tendon allograft and trimming it to approximately 6.5 cm in total length. The graft is whipstitched on both ends (FiberLoop; Arthrex, Naples, FL). At the midpoint of the graft, a single suture in a luggage‐tag conformation is placed (FiberLink; Arthrex, Naples, FL). The 2 ends of the graft can be whipstitched with different‐colored suture to make identification of the limbs easier intraoperatively (Figure 2) (Table 1).

**TABLE 1:** Pearls and Pitfalls of Arthroscopic Anterior and Posterior Capsular Augmentation With Gracilis Tendon Autograft

Pearls
Use sutures of different colors during graft preparation to facilitate easier identification intraoperatively.
Each graft should be trimmed to appropriate length (55 mm anteriorly, 65 mm posteriorly) before placement of whipstitches.
Counter‐tension should be maintained on the trailing sutures of both grafts during passage into the joint to reduce the risk of suture or graft entanglement.

Pitfalls
Entanglement of the graft or sutures while shuttling into joint can elongate procedure time.
Ensure no twisting of the graft or having too much graft enter the pilot hole, as this can result in inadequate fixation with the anchor or obscure visualization.

Similarly, preparation of the graft for anterior capsular augmentation begins with a gracilis tendon allograft being trimmed to approximately 5.5 cm. The process of graft preparation described previously is repeated, with the only difference being a slightly shorter graft being utilized for the anterior capsular augmentation.

#### Graft Shuttling and Anchor Placement

**FIGURE 3:**
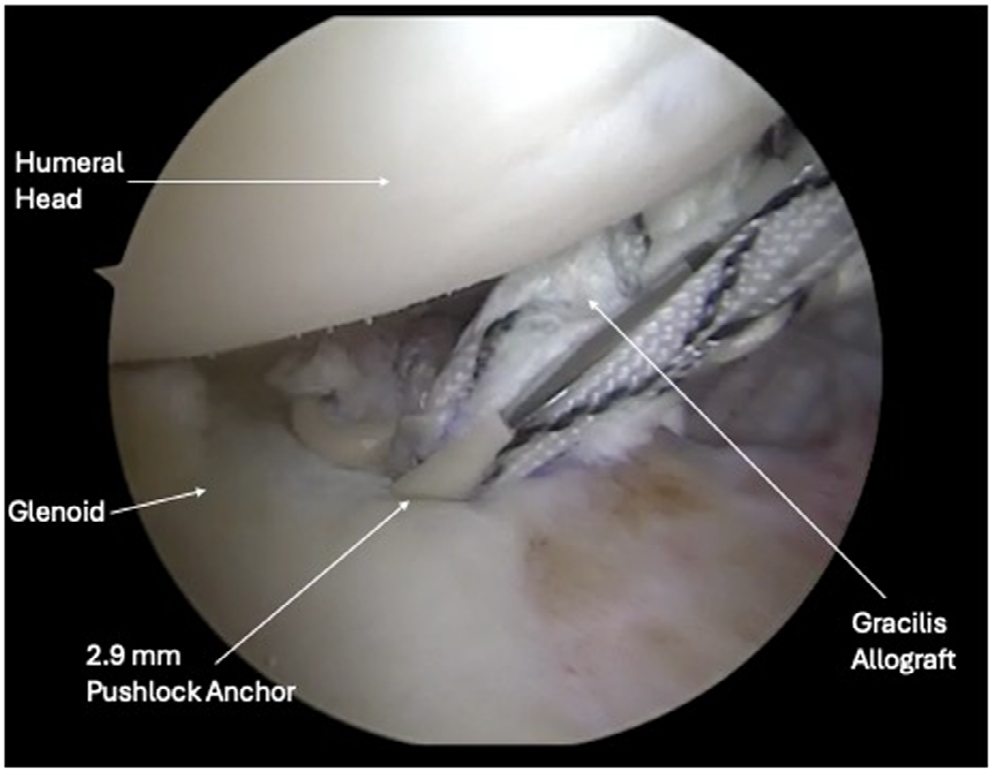
Left shoulder with the patient in the lateral decubitus position. Viewing through the posterior portal, the 2.9‐mm PushLock anchor is being placed into the inferior posterior pilot hole, loaded with the gracilis allograft.

Viewing from the anterior portal, a cannula with internal deployable wings (Gemini cannula; Arthrex, Naples, FL) is placed through the previously established posterior portal. The arthroscope is then moved to the previously established posterior portal and the working anterior portals are established. Typically, 2 cannulas (Twist‐In cannula sizes 8.25 mm × 7 cm and 6 mm × 7 cm; Arthrex, Naples, FL) are placed through the rotator interval anteriorly and utilized for both visualizing and passing graft and suture with the larger cannula being placed inferiorly. The cannulas are placed by making a small incision through the skin and then blunt dissection with the trochar to the desired position through the joint capsule within the rotator interval. The inferior anchor should be placed close to the superior boarder of the subscapularis to allow enough room in the rotator interval for a second cannula to be placed in the interval but on the same side of the biceps tendon. A drill guide for the 2.9‐mm PushLock suture anchor (Arthrex, Naples, FL) is introduced through the posterior cannula, and 2 pilot holes are drilled into the inferior‐posterior and superior‐posterior glenoid. The first PushLock anchor, loaded with the whipstitched end of the graft, is inserted into the more inferior‐posterior pilot hole, and the remnant suture is cut with an arthroscopic cutter (Figure 3). Care should be taken to avoid graft twisting or excess graft entering the pilot hole.

**FIGURE 4:**
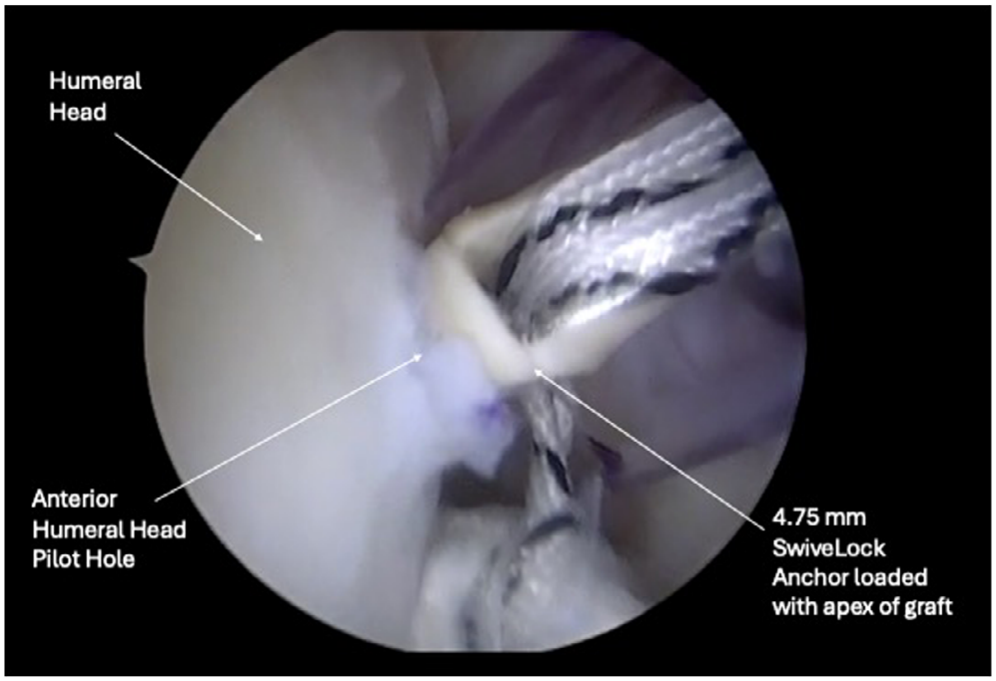
Left shoulder, with the patient in the lateral decubitus position. Viewing through the posterior portal, the 4.75‐mm SwiveLock anchor, loaded with the luggage tag stitch on the apex of the graft, is placed into the anterior humeral head pilot hole.

**FIGURE 5:**
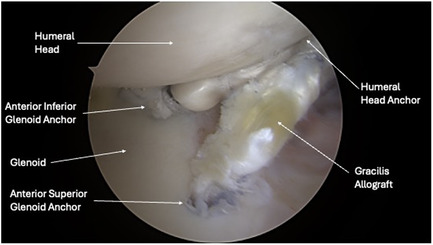
Left shoulder, viewing through posterior portal with the patient in the lateral decubitus position, the final anterior graft augmentation is seen, with a sling created by two anchors in the glenoid surface and the apex of the graft anchored to the humeral head.

The other end of the graft is placed into a second PushLock anchor and placed in a similar fashion in the posterior‐superior pilot hole. This is tensioned down in a knotless fashion using the internal mechanism within the anchor. After this, a punch is used to prepare a site in the posterior humeral head opposite and halfway between the 2 anchored ends of the graft. The luggage tag FiberLink suture at the central apex of the graft is loaded into a 4.75‐mm SwiveLock anchor (Arthrex, Naples, FL). The SwiveLock anchor is then anchored into the humeral head to complete the capsular augmentation (Figure 4). The tension of the repair is established by the premeasured length of the prepared gracilis allograft. The final construct creates a sling effect with the allograft material in a triangular shape (Figure 5).

The arthroscope is then moved to the posterior portal. Anterior augmentation is performed using the same technique, with graft ends anchored at 3‐ and 5‐o'clock positions on the glenoid and the midpoint fixed to the humeral head between these anchors. A drill guide for the 2.9‐mm PushLock suture anchor (Arthrex, Naples, FL) is introduced through the low anterior cannula, and 2 pilot holes are drilled into the inferior‐anterior and superior‐anterior glenoid. The first PushLock anchor, loaded with the whipstitched end of the graft, is inserted into the more inferior‐anterior pilot hole, and the remnant suture is cut with an arthroscopic cutter. Care should be taken to avoid graft twisting or excess graft entering the pilot hole. The other end of the graft is placed into a second PushLock anchor and placed in a similar fashion in the superior‐anterior pilot hole. This is tensioned down in a knotless fashion using the internal mechanism within the anchor. After this, a punch is used to prepare a site in the anterior humeral head opposite and halfway between the 2 anchored ends of the anteriorly placed graft. The luggage tag FiberLink suture at the central apex of the graft is loaded into a 4.75‐mm SwiveLock anchor (Arthrex, Naples, FL). The SwiveLock anchor is then anchored into the humeral head to complete the capsular augmentation anteriorly. The tension of the repair is established by the premeasured length of the prepared gracilis allograft. The final construct creates a sling effect with the allograft material in a triangular shape.

Portals are closed in standard fashion and a sterile dressing is applied.

### Postoperative Care

The operative shoulder should be kept in a shoulder immobilizer with an abduction pillow for 6 weeks after surgery except during physical therapy and home exercises. Physical therapy begins with early, controlled range of motion (ROM), starting with passive movements and progressing to active‐assisted motion (phase 1). This includes external rotation at 90° of abduction and forward flexion up to 90°, while active internal rotation is avoided for the first 8 weeks. Submaximal isometric shoulder exercises and rhythmic stabilization drills are introduced 2 months postoperatively.

From weeks 9 to 16 (phase 2), rehabilitation focuses on restoring ROM, improving joint mechanics, building strength, and enhancing neuromuscular control and proprioception. By week 16, the patient should have full, pain‐free ROM, no tenderness, and 70% strength compared with the opposite shoulder. Phase 3 emphasizes dynamic strengthening, with unrestricted activity typically allowed between 24 and 28 weeks. The goal is a stiff, stable shoulder; lack of terminal or “end‐range” external and internal rotation is considered surgical success.

## DISCUSSION

This article describes an arthroscopic anterior and posterior glenohumeral capsular augmentation technique that can be used to address multidirectional instability in the shoulder joint in a revision setting for a hypermobile patient with EDS with subscapularis insufficiency. Previously, a posterior capsular augmentation and sling technique has been described, which prevents instability and maintains good ROM.^17^ The addition of an anterior allograft sling to the previously described surgical technique can provide additional anterior stability, which is advantageous in patients with chronic subscapularis deficiency, multidirectional instability, and previously failed operations. Other techniques that could be used in patients with adequate tissue quality include subscapularis augmentation with allograft or anterior capsular reconstruction.^18^, ^19^ Pancapsular shift is another common surgical approach for these challenging patients; however, in patients with EDS, this may be ineffective due to the poor tissue quality and may lead to failure and need for revision surgery.^1^

For anterior reconstructions, many different grafts have been used, including the Achilles tendon, iliotibial band, hamstring tendon, and tibialis anterior tendon.^20^, ^21^, ^22^, ^23^ The Achilles tendon allograft can also be used in a posterior approach.^23^ Advantages of our proposed technique include combined anterior/posterior stabilization, compatibility with concomitant arthroscopy, and knotless fixation (Table 2). Additionally, we utilize allografts that have improved tissue quality compared with EDS host autograft tissue, which would be sutured during traditional capsulorrhaphy or open anterior‐inferior capsular shift surgical techniques. Potential limitations of this technique include the risk of failure at the anchor, graft, or graft‐suture interface, which may result in intra‐articular loose bodies requiring revision arthroscopy. Additionally, long‐term outcomes compared with other augmentation techniques remain limited, and the described procedure does not address cases with significant glenoid or humeral head bone loss.

**TABLE 2:** Advantages and Disadvantages of Arthroscopic Anterior and Posterior Capsular Augmentation With Gracilis Tendon Autograft

Advantages	Disadvantages
Provides circumferential (anterior and posterior) stabilization, restoring multidirectional stability	Failure at the anchor or graft‐suture interface can lead to intra‐articular loose bodies requiring further revision arthroscopy
Can be performed arthroscopically, minimizing the risks associated with similar open procedures	Long‐term outcomes compared with other augmentation techniques are limited
Utilizes allograft tissue to overcome poor native tissue quality in patients with Ehlers‐Danlos syndrome	Does not address humeral or glenoid bone loss

The posterior capsular augmentation technique has produced encouraging results in patients in restoring stability in previously unstable shoulders.^11^ This proposed anterior and posterior augmentation technique can be considered in similar patient cohorts who have multidirectional instability and subscapularis deficiency.

## DISCLOSURES

The authors (M.D.M., S.M.) declare the following financial interests/personal relationships, which may be considered as potential competing interests. M.D.M. reports a relationship with *American Journal of Sports Medicine* that includes board membership; reports a relationship with Arthrex Inc. that includes consulting or advisory; reports a relationship with Telephus that includes equity or stocks; reports a relationship with Prodigy Surgical Distribution that includes nonfinancial support. S.M. reports a relationship with Arthrex Inc. that includes consulting or advisory. The other authors (O.N.P., S.L., R.L., B.P.S.) declare that they have no known competing financial interests or personal relationships that could have appeared to influence the work reported in this article.
